# High-throughput tri-colour flow cytometry technique to assess *Plasmodium falciparum* parasitaemia in bioassays

**DOI:** 10.1186/1475-2875-13-412

**Published:** 2014-10-20

**Authors:** Regis W Tiendrebeogo, Bright Adu, Susheel K Singh, Daniel Dodoo, Morten H Dziegiel, Benjamin Mordmüller, Issa Nébié, Sodiomon B Sirima, Michael Christiansen, Michael Theisen

**Affiliations:** Department of Clinical Biochemistry, Immunology and Genetics, Statens Serum Institut, Copenhagen, Denmark; Centre for Medical Parasitology at Department of International Health, Immunology, and Microbiology and Department of Infectious Diseases, Rigshospitalet, University of Copenhagen, Copenhagen, Denmark; Noguchi Memorial Institute for Medical Research, University of Ghana, Legon, Ghana; Blood Bank KI 2034, Copenhagen University Hospital, Copenhagen, Denmark; Institute of Tropical Medicine, University of Tübingen, Wilhelmstraße 27, Tübingen, D-72074 Germany; Centre de Recherche Médicale de Lambaréné (CERMEL), Lambaréné, BP 118 Gabon; Centre National de Recherche et de Formation sur le Paludisme, Ouagadougou, Burkina Faso

**Keywords:** Malaria, *Plasmodium falciparum*, Mitotracker red, Coriphosphine-O, ADCI, Bioassay, Flow cytometry, CD45, Tri-colour

## Abstract

**Background:**

Unbiased flow cytometry-based methods have become the technique of choice in many laboratories for high-throughput, accurate assessments of malaria parasites in bioassays. A method to quantify live parasites based on mitotracker red CMXRos was recently described but consistent distinction of early ring stages of *Plasmodium falciparum* from uninfected red blood cells (uRBC) remains a challenge.

**Methods:**

Here, a high-throughput, three-parameter (tri-colour) flow cytometry technique based on mitotracker red dye, the nucleic acid dye coriphosphine O (CPO) and the leucocyte marker CD45 for enumerating live parasites in bioassays was developed. The technique was applied to estimate the specific growth inhibition index (SGI) in the antibody-dependent cellular inhibition (ADCI) assay and compared to parasite quantification by microscopy and mitotracker red staining. The Bland-Altman analysis was used to compare biases between SGI estimated by the tri-colour staining technique, mitotracker red and by microscopy.

**Results:**

CPO allowed a better separation between early rings and uRBCs compared to mitotracker red resulting in a more accurate estimate of total parasitaemia. The tri-colour technique is rapid, cost effective and robust with comparable sensitivity to microscopy and capable of discriminating between live and dead and/or compromised parasites. Staining for CD45 improved parasitaemia estimates in ADCI assay since high numbers of leucocytes interfered with the accurate identification of parasitized RBC. The least bias (-1.60) in SGI was observed between the tri-colour and microscopy.

**Conclusion:**

An improved methodology for high-throughput assessment of *P. falciparum* parasitaemia under culture conditions that could be useful in different bioassays, including ADCI and growth inhibition assays has been developed.

## Background

Malaria remains a major global health burden and efforts are being intensified to find effective vaccines and chemotherapeutics against the disease [1–3]. Both malaria vaccine and pharmacologic studies rely on bioassays to identify, choose and monitor candidate vaccines and drugs in their preclinical and clinical development. The endpoint of these bioassays is often critically dependent on accurate estimation of parasite growth dynamics, traditionally achieved by microscopical examination of Giemsa-stained slides. Alternatively, others have used the standard [^3^H]-hypoxanthine incorporation assay [4], immuno-enzymatic assays measuring proteins such as *Plasmodium falciparum* lactate dehydrogenase enzyme or the histidine rich protein-2 [5, 6] to assess parasite growth in bioassays. Microscopy remains the ‘gold standard’ method for quantifying malaria parasites and characterization of species and developmental stages, although the method is far from perfect [7, 8]. Among the major drawbacks of microscopy are that it requires well-trained microscopists and cannot be used for high-throughput experiments. In some cases results can be subjective and ambiguous even under the ‘expert’s’ eye [7, 9]. Molecular methods have been developed as an alternative to microscopy and although they offer higher sensitivity and specificity, they are as yet not robust and economical enough for most routine applications [10]. The use of radio-labelled compounds has become less popular since it could have more adverse health and environmental consequences and often requires specialised and expensive equipment set up. In recent years, sophisticated flow cytometry-based protocols that allow for high precision and more objective multi-parameter analysis of malaria parasites have been explored. These protocols primarily rely on cell permeable dyes, such as acridine orange [11], DRAQ-5 [12], ethidium bromide [13], hydroethidine [14], SYBR Green I [15, 16], hoechst [17], thiazole orange [18], SYTO-16 [19], and propidium iodide [20], that stain parasite nucleic acids within infected erythrocytes. Cell-impermeant dyes such as YOYO-1 [21, 22] or SYTOX-Green [23] have also been employed. Coriphosphine O (CPO) is a cell membrane permeant metachromic dye which stains both deoxyribonucleic acid (DNA) and ribonucleic acid (RNA) with the emission of strong green and red fluorescence, respectively, upon excitation and has been used to analyse reticulated platelets [24]. It is excitable at 488 nm, making it suitable for most argon ion lasers found in standard flow cytometers, and exhibits a large Stokes shift upon excitation when bound to nucleic acids, making it a potentially useful dye for high-resolution parasitaemia estimations in bioassays. However, nucleic acid staining dyes are generally poor at distinguishing between live and dead cells since they can also bind residual DNA and/or RNA from dead or compromised parasites as has been indicated using hoechst 33342, thiazole orange and DiIC_1_-5 [25]. Jogdand *et al.* [26] recently developed a flow cytometry method based on the mitotracker red CMXRos dye, which preferentially stains only live cells with viable mitochondrial membrane potential to quantify *P. falciparum*-infected erythrocytes. Although mitotracker red staining was very efficient in late parasite developmental stages such as trophozoites and schizonts with well-developed mitochondria, the resolution between early-ring stages and uninfected red blood cells (uRBCs) was rather reduced, most likely due the small mitochondrion present in rings [26]. In bioassays that involve leucocytes, such as the antibody-dependent cellular inhibition (ADCI) assay, or in animal and human studies where *Plasmodium* infection can lead to premature release of nucleated erythrocyte precursors [23], neither nucleic acid nor mitochondria potential dyes alone or in combination may yield precise parasitaemia estimates. This is because most of these cells possess mitochondria and/or nucleic acids and discrimination based on size alone may not sufficiently exclude their confounding effect on accurate parasitaemia estimation [15, 23, 27]. Here, a new, rapid and robust three-parameter flow cytometry method for enumeration of *P. falciparum*-infected erythrocytes based on the nucleic acid staining dye-CPO, the mitochondrial membrane potential dye-mitotracker red CMXRos and the pan-leucocyte marker-CD45 was developed. The method was used in estimating the specific growth inhibition index (SGI) in the ADCI assay and was found to be in strong agreement with microscopy, the current gold-standard technique.

## Methods

### Ethics statement

Ethical approval for the Ghanaian samples in the study was given by the Institutional Review Board of the Noguchi Memorial Institute for Medical Research of the University of Ghana, Accra, Ghana. Parents and guardians gave written informed consent before their children were enrolled into the study. Ethical approval for Danish blood donor samples was given by the Scientific Ethics Committee of Copenhagen and Frederiksberg, Denmark. These were individuals residing in central Copenhagen and provided written consent for a small amount of their blood stored, anonymously, and used for research purposes.

### Plasmodium falciparum NF54 culture

*Plasmodium falciparum* strain NF54 was cultured as described elsewhere [28]. Briefly, parasites were maintained in culture using 2.5% haematocrit of human blood group O + in parasite growth medium (PGM) consisting of RPMI 1640 (Lonza, USA) supplemented with 0.5% Albumax, 25 mM HEPES, 2 mM L-glutamine, 24 mM NaHCO_3_, 25 μM gentamicin and 10% (v/v) heat-inactivated human blood group AB serum. Culture was maintained at 37°C in 25-sq cm flasks after gassing with a gas mixture containing 5% O_2_, 5% CO_2_ and 90% N_2_. For the staining assays, asynchronous parasite cultures were used while successive treatment with 5% D-sorbitol [29] was used to synchronise cultures for the antibody dependent cellular inhibition assay (ADCI) assay. To obtain high-parasitaemia cultures for staining assays without driving parasites into crisis state or gametocytogenesis, cultures were double synchronized by D-sorbitol treatment and enrichment for matured stage parasites by magnetic separation (Miltenyi Biotec) after 70% Percoll treatment as described [30]. High-parasitaemia cultures were maintained at low haematocrit (0.5%) with three media changes per week.

### Microscopy

Microscopy analysis of culture parasitaemia was performed in thin blood smears fixed with 100% methanol and stained with Giemsa (Merck Co, Germany) in a 1:10 dilution in RPMI 1640 (Lonza, USA). After 10-min incubation at room temperature, slides were washed with distilled water and dried at room temperature. Parasitaemia was estimated by counting approximately 5,000 erythrocytes under oil immersion (×100) objective lens.

### Test antibodies

Total immunoglobulin (Ig) G was purified from plasma samples as previously described [31]. The negative and positive control IgG used in the ADCI assays were pooled IgG from malaria-naïve Danish individuals (PNIG) and pooled IgG from hyper-immune African adults (PHIG), respectively [26, 31]. The test samples were IgG purified from 19 Ghanaian children (age range one to 12 years) naturally exposed to malaria [32].

### Intra-erythrocytic parasite staining

To obtain the optimal concentration of CPO for staining parasites, 3 μl *P. falciparum* NF54 asynchronous culture at ~13% parasitaemia (microscopical estimation) was added to each well of a 96-well round-bottom plate (Nunc, Roskilde, Denmark) containing 100 μl PGM and centrifuged (Sigma 3-16 K, Germany) at 770 × g for 5 min at room temperature (rt) to harvest cells. Cell pellets were resuspended in 100 μl of PGM containing 100 nM, 20 nM, 5 nM and 2.5 nM CPO (Tokyo Chemical Industry Co, Japan), respectively, in duplicate wells and incubated in the dark at 37°C for 30 min. Cells were washed (centrifugation at 770 × g for 5 min at 5°C) twice, each with 100 μl PGM. Pellets were resuspended in 200 μl PGM for analysis by flow cytometry to identify the best CPO concentration for staining parasites.

### Mitotracker red-CPO dual staining of parasite

CPO was combined with 5 μM of mitotracker red dye [26]. The double staining protocol was assessed using a *P. falciparum* NF54 asynchronous culture at ~4.0% parasitaemia. Here, 3 μl of parasite culture were co-stained with 100 μl of the CPO-mitotracker red mix in PGM and incubated at 37°C for 30 min in the dark. Cells were then washed twice as described above and resuspended in 200 μl PGM for counting in the flow cytometer.

### Drug treatment

To investigate the dynamics of CPO and mitotracker red dual-colour staining, *P. falciparum* NF54 asynchronous culture at 2% parasitaemia and 2.5% haematocrit was treated with 10 nM atovaquone/1 μM proguanil for up to 48 hours to depolarize the mitochondria membrane potential [26, 33]. Parasitaemia prior to drug treatment (0 hrs) and at 24 hrs and 48 hrs post-treatment was estimated by flow cytometry using the CPO-mitotracker red dual-colour staining protocol as described above. Additionally, the same samples were analysed by confocal microscopy.

### Tri-colour staining and gating strategy

To develop the tri-colour staining protocol that enables exclusion of leucocytes from the assay, *P. falciparum* NF54 asynchronous culture of ~15% parasitaemia and 2.5% haematocrit was mixed with ~1% of human monocyte isolated with the EasySep™ Human Monocyte Enrichment Kit (STEMCELL™ Technologies, France). The resulting mixture was then stained with the CPO-mitotracker red dual-colour staining protocol in a 96-well round-bottom plate. Control wells contained a mixture of uRBCs and ~1% of human monocyte. Following the 30 min of CPO-mitotracker red incubation in the dark at 37°C, the plate was centrifuged at 770 × g for 5 min at 5°C to remove excess dye and cells were washed once in 100 μl PGM. Five μl of mouse anti-human CD45 mAb (clone H130), PerCP conjugate (Invitrogen™, USA) was added and cells were incubated at 4°C for 10 min resulting in the tri-colour staining protocol. Excess CD45 mAb was removed by centrifugation and the cells washed once with 100 μl PGM. Cells were resuspended in 200 μl PGM for counting in the flow cytometer.

### Tri-colour staining reliability

*Plasmodium falciparum* NF54 asynchronous culture of ~30% parasitaemia and 2.5% haematocrit was mixed with ~26% of human monocyte isolated with the EasySep™ Human Monocyte Enrichment Kit (STEMCELL™ Technologies, France). The resulting mixture was serially diluted two-fold using erythrocytes at 2.5% haematocrit in PGM in a 96-well round-bottom plate so that the final theoretical parasite and monocyte proportions were 0.0037 and 0.0032%, respectively. Microscope slides were prepared for each well and stained with Giemsa to estimate the ‘expected’ parasitaemia by microscopy. The remaining cells were then stained using the tri-colour staining technique and analysed in the flow cytometer. Monocyte counting by microscopy was not possible after the serial dilutions therefore the ‘expected’ monocyte proportion was estimated from the dilution factor and compared with the tri-colour measurements.

### Flow cytometry analysis

Flow cytometry data acquisition was done on Beckman Coulter cytometer (Cytomics FC500 MPL) using the argon ion laser (488 nm). The detectors of forward scatter, side scatter, FL1 (for detection of DNA bound CPO signal), FL3 (for detection of mitotracker red) and FL4 (for detection of CD45-PerCP) were set in logarithmic mode. The excitation wavelength and the detector filters of CPO, mitotracker red and CD45-PerCP are, respectively, 500 nm/525 BP, 579 nm/620 BP and 488 nm/ 675 BP. The machine was set up by initially running unstained cells and cells that had been stained separately with each fluorochrome. Appropriate electronic colour compensations were adjusted between the three dyes. DNA-bound CPO florescence in FL1 was preferred to RNA-bound CPO signal in FL2 for parasite quantification to minimize the signal overlap with mitotracker red in FL3, thereby making electronic colour compensation much simpler between the two dyes. Also, DNA is fairly constant compared to RNA, which may vary widely depending on the state of the parasite. The average data acquisition speed was 500 events per sec for 1 min with a minimum of 30,000 events or more than 300 infected red blood cells (iRBCs) counted [26] and analysed with Kaluza Analysis Software (Version 1.3). In the tri-colour staining, events were first gated according to their FSC/SSC profile to exclude doublets and debris. A second gate in the dot plot SSC/FL4-CD45-PerCP was set up to exclude leucocytes (monocytes) and the CD45-PerCP negative cell population was then carried into the next level of analysis where they were displayed in SSC/FL1-CPO dot plot to identify all iRBCs (total parasitaemia). Finally, the positive events were then displayed in SSC/FL3-mitotracker red dot plot to estimate iRBCs with viable mitochondrial membrane potential and those negative for mitotracker red signal (dead and/or compromised cells).

### Antibody-dependent cellular inhibition assay

The ADCI assays were performed as previously described [26], with slight modifications. Briefly, about 2 × 10^5^ monocytes/well were selected by adherence to a 96-well flat-bottom culture plates (Nunc, Roskilde, Denmark) after a 2-hr incubation of two million PBMCs/well at 37°C and 5% CO_2_ in monocyte medium (RPMI medium containing 5% NHS, 1% glutamine and 1% penicillin-streptomycin). Highly synchronized parasite culture, at late schizont stage of 0.5% parasitaemia and 2.5% haematocrit were added, 100 μl/well. Control and test antibodies were added to respective wells at 1 mg/ml and the total volume adjusted to 200 μl with PGM for wells containing uRBCs and blank controls. Control wells included: (i) parasite culture without monocytes; (ii) culture with monocytes but without IgG; (iii) culture with pool of naïve Danish IgG (PNIG); (iv) culture with monocytes and PNIG; (v) culture with pool of hyper immune African adult IgG (PHIG); and, (vi) culture with monocytes and PHIG. At 48 hrs and 72 hrs respectively, an additional 50 μl of PGM was added to each well and after 96 hrs the assay was stopped and the final parasitaemia determined by microscopy and flow cytometry using the novel tri-colour method. The 19 different test samples were tested on different days and the corresponding microscopy slides for each sample were also obtained for parasitaemia determination. The SGI estimating the parasite growth inhibition due to the effect of antibodies co-operating with monocytes (MN) was calculated as follows: SGI = 100 × (1 - (% parasitaemia with MN and test antibodies/% parasitaemia test antibodies)/(% parasitaemia with MN and PNIG/% parasitaemia PNIG)).

### Statistical analysis

The two-way ANOVA for overall comparison and subsequent pairwise comparisons corrected with the Bonferroni method were used to test differences between the parasitaemia estimated by CPO and mitotracker red during the drug treatment experiment. Linearity in the estimation of parasitaemia and monocyte proportions, respectively, by the tri-colour staining technique was determined by Pearson’s *r* and linear regression. The Bland-Altman analysis was used to compare biases between SGI estimated by the tri-colour staining technique, mitotracker red and by microscopy. All statistical analyses were performed using GraphPad Prism vs.5.04 for Windows, (GraphPad Software, USA).

## Results

### Determination of optimal CPO concentration for staining *Plasmodium falciparum*

**Figure 1:**
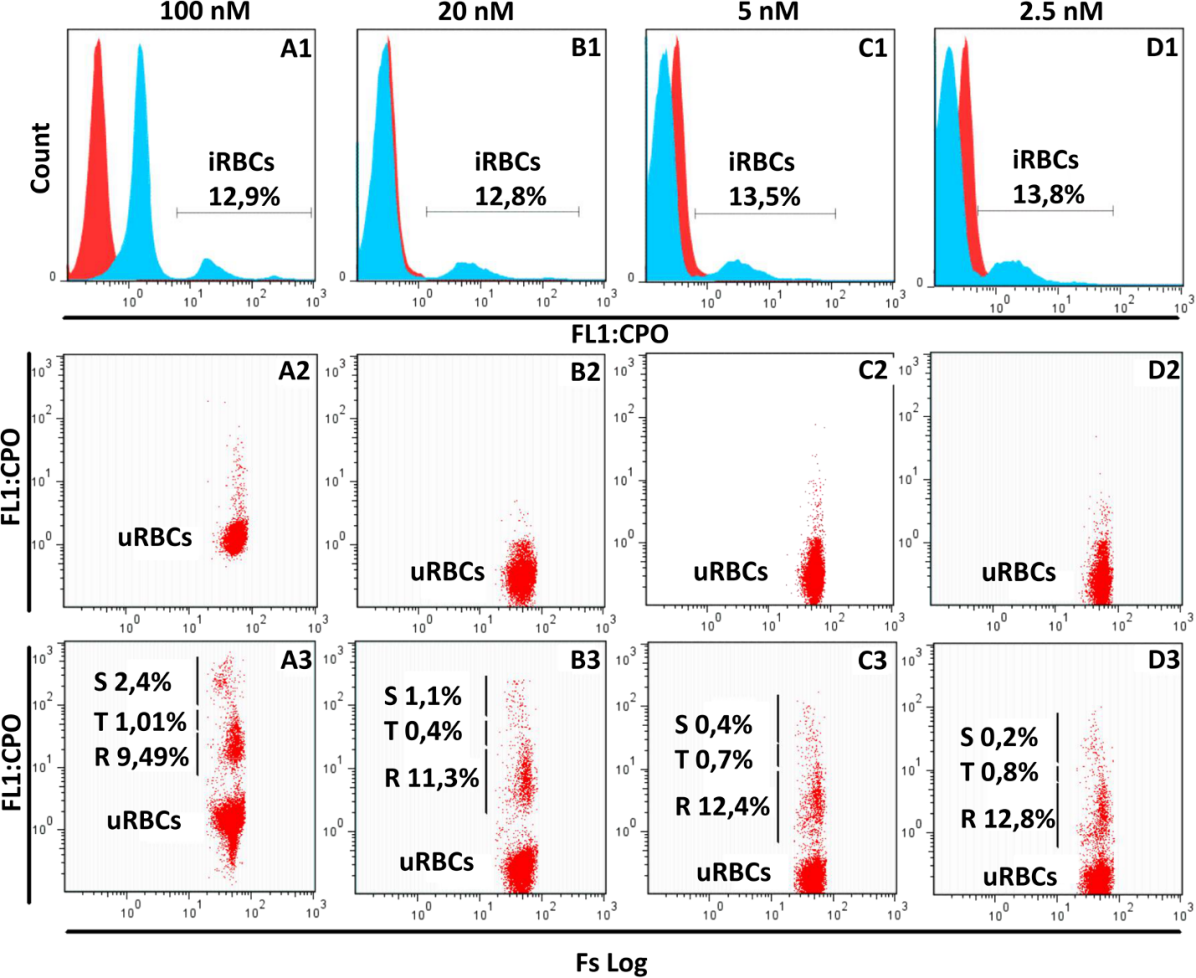
**Optimization of coriphosphine O staining concentration.** *Plasmodium falciparum* NF54 asynchronous culture at ~13.0% parasitaemia, 2.5% haematocrit was stained with different concentrations of coriphosphine-O in separate wells shown column-wise as: **(A1, A2, A3)** for 100 nM; **(B1, B2, B3)** for 20 nM; **(C1, C2, C3)** for 5 nM and **(D1, D2, D3)** for 2.5 nM. The top row **(A1, B1, C1, D1)** is a histogram showing a test sample (in blue) with infected red blood cells (iRBCs) and a control sample (in red) of uninfected red blood cells (uRBCs). The CPO staining at 20 nM gave the closest total parasitaemia (12.8%) to the microscopy reading and the best separation between iRBCs and uRBCs. The middle row **(A2, B2, C2, D2)** is a dot plot (CPO *versus* FS Log) of CPO staining of the uRBC control sample for each concentration. The 20 nM concentration also gave the least background of non-specific staining of uRBCs. The bottom row **(A3, B3, C3, D3)** shows a dot plot (CPO *versus* FS Log) of the iRBC sample with the proportions of the various parasite stages indicated; **S**: schizonts; **T**: trophozoites; **R**: rings.

*Plasmodium falciparum* NF54 asynchronous culture at ~13.0% parasitaemia (microscopy estimate) and 2.5% haematocrit was stained with CPO at various concentrations ranging from 100 nM to 2.5 nM (Figure 1). All CPO concentrations gave a parasitaemia close to the count obtained by microscopy. Twenty (20) nM CPO was chosen for subsequent experiments since it best discriminated between iRBCs and uRBCs without measurable background staining. In addition, the clearest discrimination of the different parasite developmental stages was observed at the 20 nM CPO staining concentration (Figure 1; Panel B1, B2, B3).

### Estimation of live *Plasmodium falciparum*by CPO-mitotracker red dual-staining

**Figure 2:**
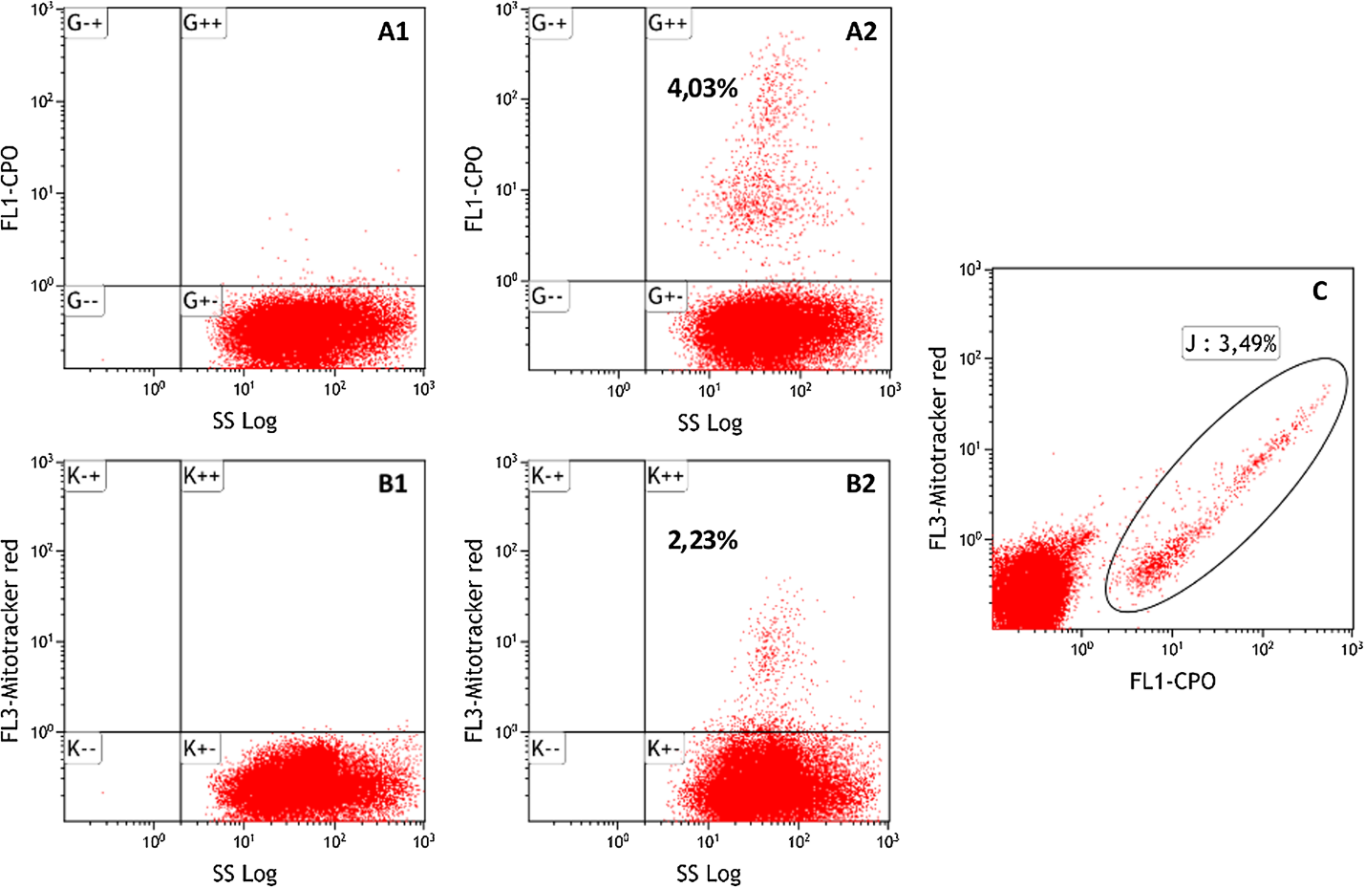
**CPO gives a better distinction between early rings and uninfected erythrocytes compared to mitotracker red.** *Plasmodium falciparum* NF54 asynchronous culture at ~4.0% parasitaemia (microscopy estimate), 2.5% haematocrit was co-stained with 20 nM CPO and 5 μM. Dot plots of SS Log/FL1-CPO for uRBCs **(A1)** and iRBCs **(A2),** respectively, are shown. There is a clear distinction between iRBCs carrying early ring parasite stages and uRBCs. Also, dot plots of SS Log/FL3-mitotracker red for uRBCs **(B1)** and iRBCs **(B2),** respectively, are shown. There is a poor distinction between iRBCs carrying early rings parasite stages and uRBCs. Quadrants are labeled ‘G’ for CPO and ‘K’ for mitotracker red plots respectively. The positive (+) and negative (-) signs denote the presence or absence respectively of a given dye signal or cell attribute in the quadrant. **(C)** Plot of FL1-CPO/FL3-mitotracker red. Infected RBCs containing live parasites are identified as mitotracker red-CPO double positive cells (3.49%).

CPO (20 nM) together with mitotracker red (5 μM) [26] was used to stain *P. falciparum* NF54 asynchronous culture at ~4.0% parasitaemia (microscopy estimate) and measured by cytometry. Applying the CPO stained uRBC control (Figure 2, Panel A1) gating to the test sample, there was a more distinct separation between uRBCs and iRBCs allowing for a much precise total parasitaemia (live and dead or compromised combined) estimation (Figure 2, Panel A2). However, when the mitotracker red-stained uRBC control (Figure 2, Panel B1) gating was applied to the test sample, the parasitaemia was underestimated (Figure 2, Panel B2) as a consequence of the poor distinction between uRBCs and early ring stages of the parasite. Thus, a strategy for estimating live parasitaemia was achieved by identifying cells that were CPO and mitotracker red double positive estimated as 3.49% (Figure 2, Panel C).

### Compromised parasites stain weakly with mitotracker red

**Figure 3:**
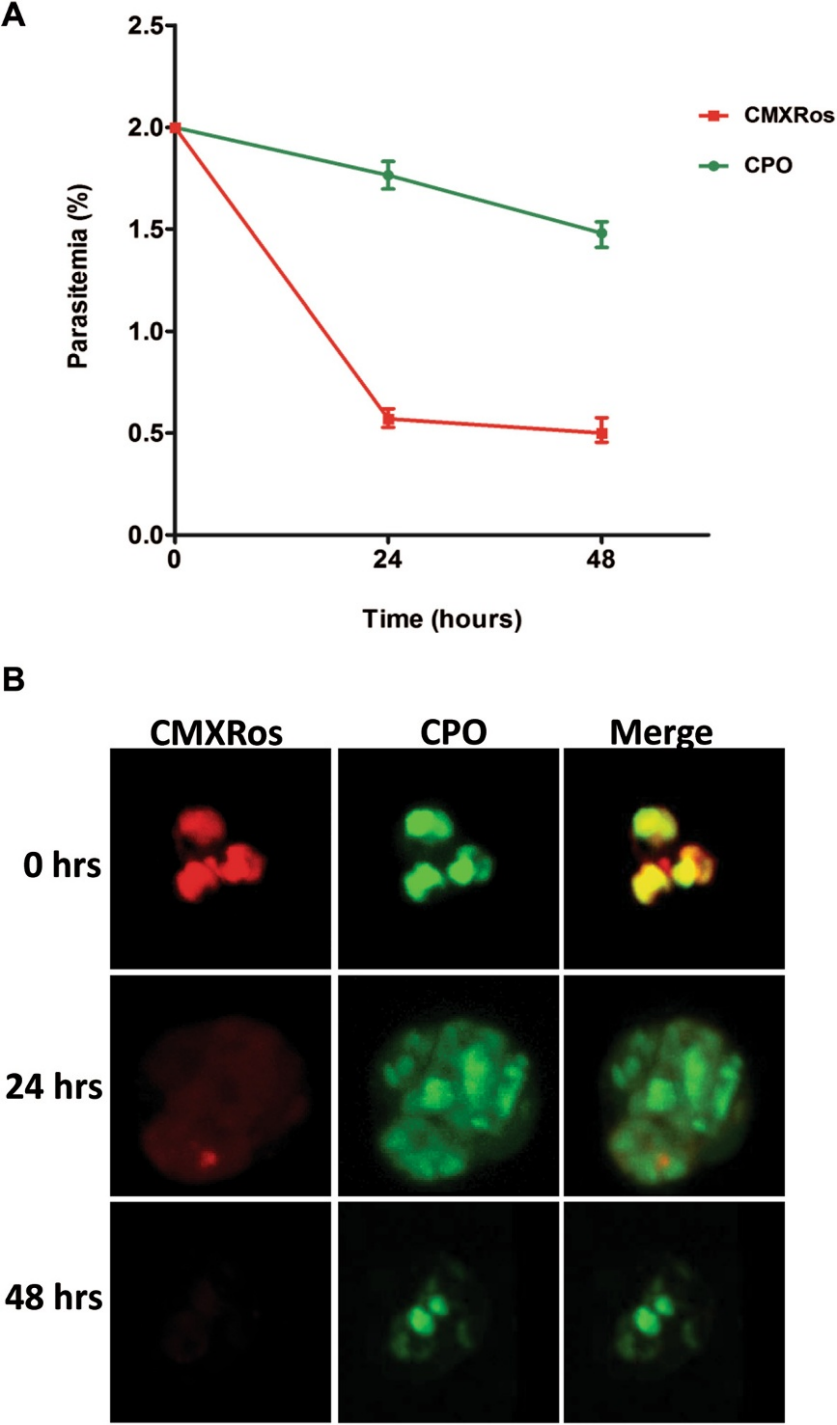
**Dynamics of mitotracker red and CPO staining of compromised parasites.** *Plasmodium falciparum* NF54 asynchronous culture at 2% parasitaemia was treated with 20 nM atovaquone/1 mM proguanil to induce compromised state parasites. **A)** Flow cytometry parasitaemia estimate by staining individually with mitotracker red (5 μM) and CPO (20 nM) at 0 hrs, 24 hrs and 48 hrs post treatment. Values are plotted ± standard deviation for triplicate wells. **B)** Representative Z-stack images (interval = 0.2 μm) by mitotracker red-CPO staining of culture corresponding to 0 hrs, 24 hrs and 48 hrs post treatment. Each row represents a sampling time and shows, from left to right mitotracker red staining, CPO staining and the merged image.

The robustness of the developed strategy to distinguish between compromised (‘crisis’ or dead) and a healthy (live) *P. falciparum* parasite was further investigated by both flow cytometry and confocal imaging analyses. After 24 hrs of atovaquone and proguanil treatment designed to induce compromised state parasites, the initial 2% parasitaemia dropped sharply to 0.5% by mitotracker red estimate and moderately (1.8%) by CPO estimate (Figure 3A). No further decrease in parasitaemia estimate was observed by mitotracker red staining while the CPO estimate showed a marginal decrease to 1.5% after 48 hrs (Figure 3A). The parasitaemia estimated by CPO and mitotracker red in the drug treatment assay were significantly different (*p* < 0.0001, Two-way ANOVA). At 24 hours and 48 hours post treatment, parasitaemia estimated by CPO was significantly higher than by mitotracker red (*p* < 0.0001, Bonferroni *post hoc* test). Confocal imaging analyses coinciding with the various sampling time for the flow cytometry indicated a drastic loss of mitotracker red signal 24 hours post treatment while CPO signal only showed a gradual decrease (Figure 3B).

### Tri-colour staining and strategy of gating

**Figure 4:**
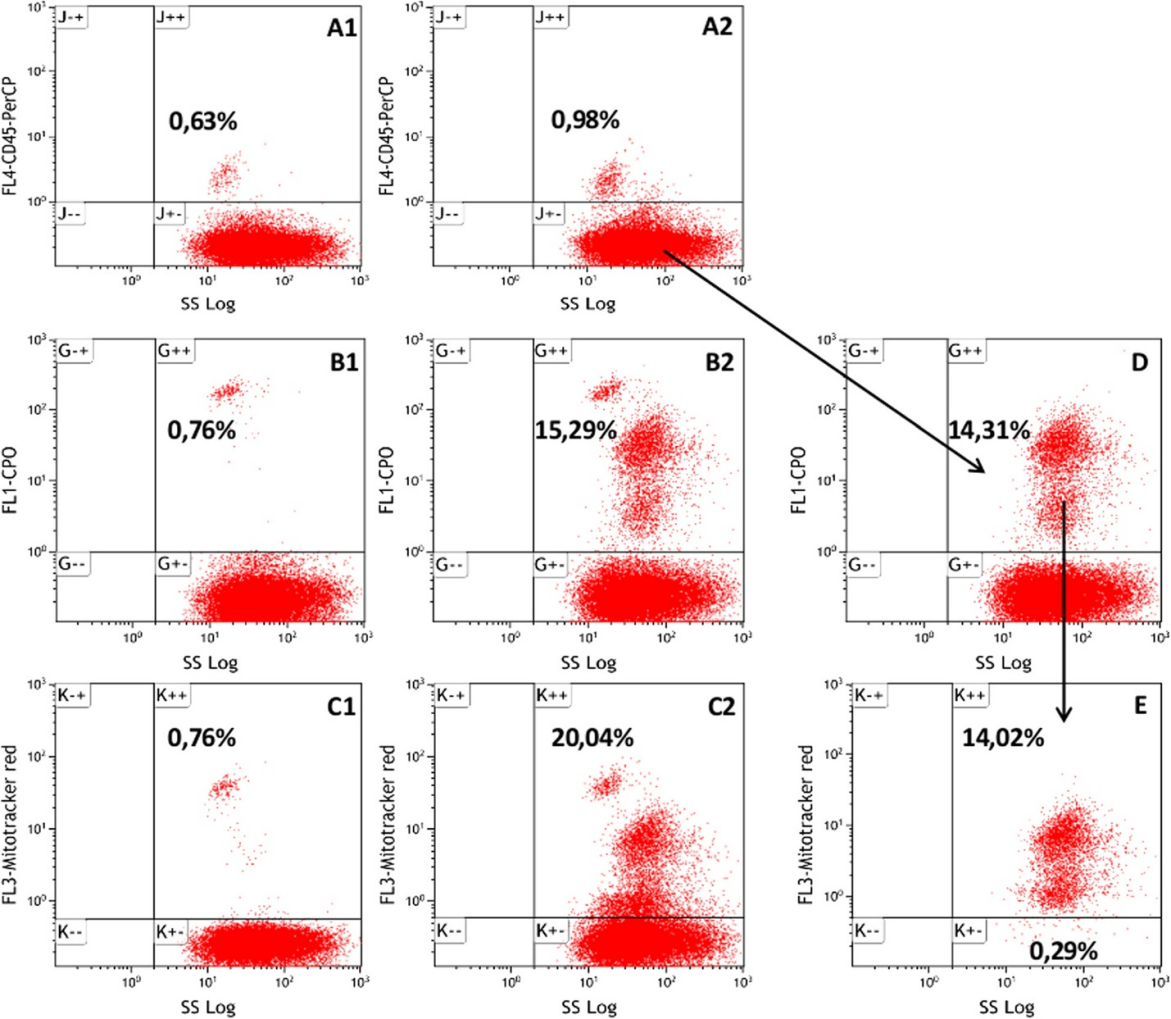
**Gating strategy for the tri-colour technique.** *Plasmodium falciparum* NF54 asynchronous culture of ~15% parasitaemia and 2.5% haematocrit was mixed with ~1% human monocyte and stained by the tri-colour technique. Panels **A1, B1** and **C1** are dot plots of SS Log/FL4-CD45-PerCP, SS Log/FL1-CPO and SS Log/FL3-mitotracker red, respectively, for the control sample containing only monocytes and uRBCs. These were used to set the threshold of positivity for the various dyes. Panel **A2** is a dot plot of SS Log/FL4-CD45-PerCP of the test sample to identify and exclude monocytes (CD45 positive cells). Panels **B2** (SS Log/FL1-CPO) and **C2** (SS Log/FL3-mitotracker red) are dot plots for the same test sample without the exclusion of the monocyte population. The proportion of CD45 negative populations (RBCs) from **A2** that were infected with *P. falciparum* are shown by the SS Log/FL1-CPO dot plot (Panel **D**). This population includes live and dead (and/or compromised) cells (i.e. total parasitaemia). Live and dead cells from the CPO-positive events were then distinguished in SS Log/FL3-mitotracker red dot plot (Panel **E**). Quadrants are labeled ‘J’ for CD45-PerCP, ‘G’ for CPO and ‘K’ for mitotracker red plots respectively. The positive (+) and negative (-) signs denote the presence or absence respectively of a given dye signal or cell attribute in the quadrant.

The tri-colour staining technique was successfully achieved by the addition of the mouse anti-human CD45 mAb conjugated to PerCP for leucocytes quantification. Leucocytes contain both nucleic acids and mitochondria and may confound parasitaemia estimates when they are present together with iRBCs in bioassays. An initial gate (FSC/SSC), of all events was defined and doublets and debris were excluded. Secondly, a dot plot SS Log/FL4-CD45-PerCP (Figure 4, Panel A1) was made for the control sample, which contained only monocytes and uRBCs. Corresponding dot plots for SS Log/FL1-CPO (Figure 4, Panel B1) and SS Log/FL3-mitotracker red (Figure 4, Panel C1) were made for the same control well to identify where monocytes are localized in each plot. In the test sample, monocytes were first excluded from total RBCs as CD45-positive cells which were estimated to be 0.98% (Figure 4; Panel A2). Dot plots of SS Log/FL1-CPO (Figure 4; Panel B2) and SS Log/FL3-mitotracker red (Figure 4; Panel C2) without the exclusion of the monocyte population demonstrate their confounding effect in CPO and mitotracker red parasitaemia estimations, respectively. After monocyte exclusion, a dot plot of SS Log/FL1-CPO was made to identify all iRBCs (total parasitaemia) (Figure 4D). Finally, the CPO-positive events were then displayed in SS Log/FL3-mitotracker red dot plot to identify iRBCs with viable mitochondrial membrane potential (live cells) and those negative for mitotracker red signal (dead and/or compromised cells) which were estimated to be 14.02 and 0.29%, respectively (Figure 4E).

### Tri-colour staining reliability in parasite and monocyte estimations

**Figure 5:**
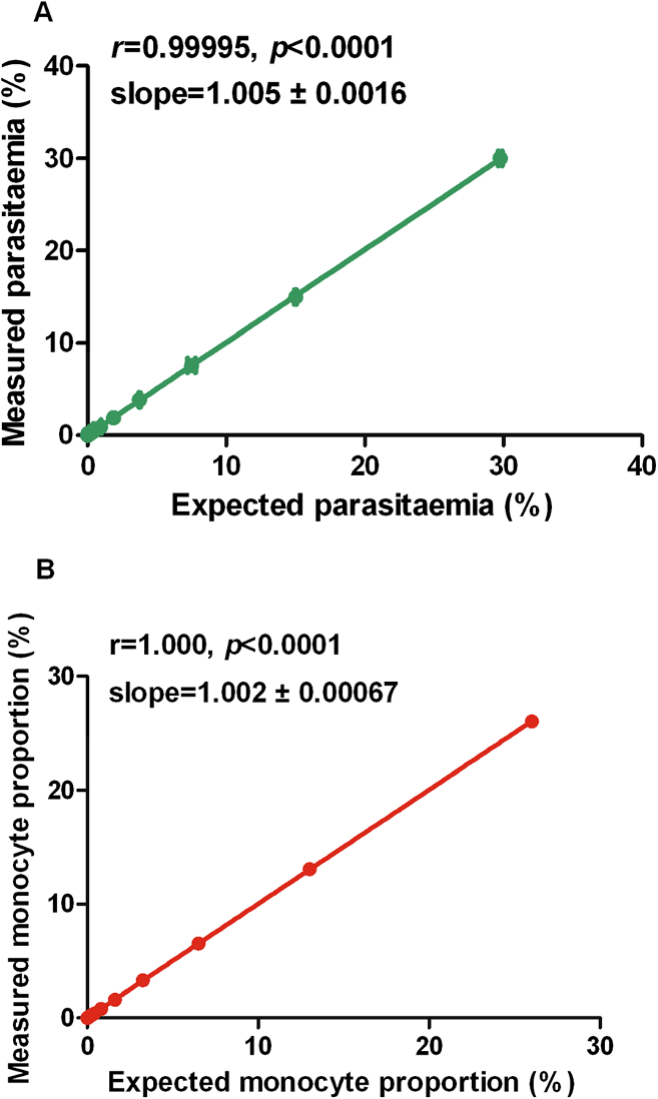
**Linearity of parasitaemia and monocyte proportions estimations by the tri-colour technique.** *Plasmodium falciparum* NF54 asynchronous culture of ~30% parasitaemia and 2.5% haematocrit was mixed with ~26% human monocyte and titrated two-fold with 2.5% haematocrit. Plots of: **A)** measured parasitaemia by the tri-colour technique *versus* expected parasitaemia by microscopy and **B)** measured monocytes proportions by the tri-colour technique *versus* expected theoretical monocyte proportions from serial dilutions are shown, respectively. There was a strong linearity in the estimation of both monocyte proportions (r = 1.00, *p* < 0.0001) and parasitaemia (r = 0.99995, *p* < 0.0001). All values are plotted ± standard deviation for triplicate wells.

The tri-colour technique could reliably estimate leucocytes and parasites separately from a mixture containing both and the proportions were comparable to the expected. There was a strong linear relationship (r = 0.99995, *slope = 1.005 ± 0.0016*) between the microscopy and the tri-colour technique estimates of parasitaemia (Figure 5). Similarly, a strong linear relationship (r = 1.00, *slope = 1.002 ± 0.00067*) was shown for the estimation of monocyte proportion by the tri-colour method when compared with the theoretical expected proportions from the dilution (Figure 5). From the serial dilutions, the lower limit for accurate parasitaemia and monocyte detection by the tri-colour was ≥0.008 and ≥0.006%, respectively.

### Tri-colour staining technique application in the ADCI assay

**Figure 6:**
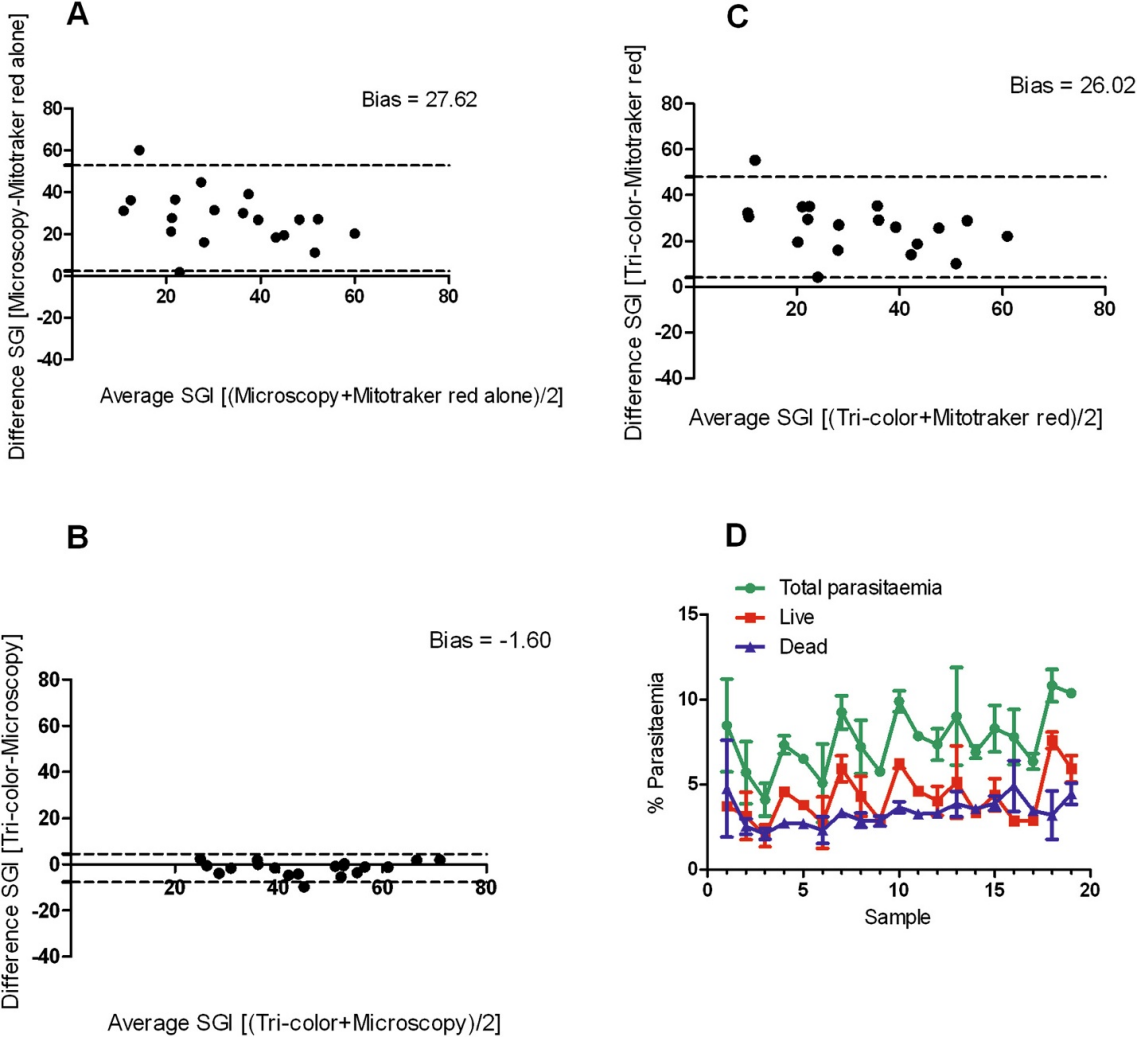
**SGI estimation agreement analysis and status of parasites in the ADCI assay.** Nineteen (19) different IgG samples from Ghanaian children were tested in the ADCI assay on different days. Bland-Altman agreement statistical test between SGI estimated by: **A)** Microscopy and mitotracker red alone staining (bias = 27.62), **B)** Tri-colour technique and microscopy (bias = -1.60) and **C)** Tri-colour technique and mitotracker red alone staining (bias = 26.02). The upper and lower 95% confidence limits of the bias between respective methods being compared are indicated as two dotted lines. **D)** Tri-colour estimation of total parasitaemia, live and dead (and/or compromised) parasites for each of the 19 samples tested in the ADCI assay. Values are plotted ± standard deviation for duplicate wells. SGI: specific growth inhibition index; ADCI: antibody dependent cellular inhibition.

The tri-colour staining method developed was applied to estimate parasitaemia for the calculation of specific growth inhibition index (SGI) of IgG samples from 19 Ghanaian children on different days. The corresponding parasitaemia estimated by microscopy examination of Giemsa-stained slides was also determined and SGI calculated. The largest bias in SGI was observed when microscopy and mitotracker red alone were compared (bias = 27.62) (Figure 6A), while agreement between microscopy and the tri-colour method (bias = -1.60) was close (Figure 6B). The bias between the tri-colour method and mitotracker red alone was large (bias = 26.02). Generally, SGI estimated by microscopy and the tri-colour methods were systematically higher than the mitotracker red estimate (Figure 6A and 6C). However, microscopy based SGI estimates were slightly higher than the tri-colour estimates (Figure 6B). The tri-colour could reliably estimate the proportions of total parasitaemia that were alive or dead (and/or compromised) in the ADCI assay (Figure 6D). The percentage of live parasitaemia was estimated as mitotracker red and CPO double positive cells after the exclusion of monocytes by CD45 gating in the tri-colour. The patterns of total and live parasitaemia reflect day-to-day variability in culture growth in the assay.

## Discussion

A high-throughput, three-parameter flow cytometry technique which combines the nucleic acid staining dye-CPO, the mitochondrial membrane potential dye-mitotracker red and a monoclonal antibody against the pan-leucocyte marker CD45 to assess malaria parasite in bioassays was developed. The technique is capable of distinguishing between different *P. falciparum* developmental stages, iRBCs containing live or dead (and/or compromised) parasites, uRBCs, and human leucocytes. The method compares well with microscopy in sensitivity and is very simple and fast requiring less than one hour for completion and can be carried out in a 96-well plate format allowing for applications that require medium to high throughput. The reagents used can be obtained at a relatively low cost and are compatible with standard flow cytometers equipped with a 488 nm argon ion laser that are found in many common laboratories. The reagent cost for the assay was estimated to be 0.12 USD per sample. In bioassays that do not involve leucocytes such as the growth inhibition assay (GIA); the technique could be applied in the dual-colour state without the CD45 marker, while the tri-colour state could be applicable to cell-mediated bioassays such as the ADCI assay.

The mitotracker red dye has a unique property of binding covalently to polarized mitochondria membrane present in live cells thus making it suitable for quantification of live parasites in bioassays. A flow cytometry technique based on this property was successfully developed and applied to the ADCI assay to estimate SGI of antibody preparations [26]. However, a common limitation of *P. falciparum* staining with mitotracker red as demonstrated here (Figure 2), is the poor resolution between uRBCs and early ring stages of the parasites. The less dense mitochondria of early ring stage parasites often results in a weak mitotracker red signal that make iRBCs containing early rings to overlap with uRBCs in the same quadrant in the mitotracker red *versus* SS-Log plot. Consequently, the true parasitaemia is underestimated since iRBCs are erroneously enumerated as uninfected. The present technique resolved this limitation by incorporating the nucleic acid staining dye CPO. Staining of *P. falciparum* cultures with CPO showed a distinct separation between uRBCs and RBCs infected with early ring stages of the parasite. CPO emits very little or no fluorescence upon excitation when unbound in non-nucleated cells such as RBCs but gives strong green emission when bound to DNA. This low background allowed for a much clearer separation between uRBCs and iRBCs observed. The mammalian-matured RBC lacks both nucleus and mitochondria, adaptive features thought to help limit the production of reactive oxygen species in such high-haem and sugar-rich environment [34]. Therefore CPO binding to intra-erythrocytic nuclear material could be used to estimate total parasitaemia (i.e., live and dead or compromised) with a much higher resolution compared to mitotracker red staining (e.g., Figure 2; Panels A2 and B2). Both flow cytometry and confocal imaging analyses of CPO and mitotracker red stained compromised state *P. falciparum* showed that mitochondrial membrane potential is rapidly lost when parasites are in ‘crisis’ but DNA may linger much longer. This may result in over-estimation of the true parasitaemia in flow cytometry techniques that employ only nucleic acid staining dyes, such as thiazole orange [18] and hydroethidine [14] which have been shown to stain both live and dead parasites [35]. In several bioassays involving *P. falciparum,* the active agents induce compromised state parasites in the killing process [36, 37], which are difficult to quantify by microscopy or distinguish from live parasites by most available flow cytometry methods [35]. Here, by initially identifying CPO positive cells, the total parasitaemia can be estimated before the proportion having active mitochondria membrane potential (i.e., live parasites) quantified by mitotracker red gating.

Previous flow cytometry techniques that quantify malaria parasites in cell-mediated bioassays, especially the ADCI assay, exclude detached leucocytes from parasitaemia estimates based on size differences [26, 38]. The inclusion of CD45 mAb in the current method provides a more empirical way of reliably identifying and excluding leucocytes that may confound parasitaemia estimations. However, the tri-colour technique had a lower limit of accurate parasitaemia detection of ≥0.008% which makes it less sensitive compared to real-time PCR [39] but similar to microscopy [40]. Hence, it may not be suitable for bioassays where extremely low parasitaemia are involved or in such situations, increased replicates of uRBC controls are recommended. On the other hand, it had a much lower limit of accurate leucocyte detection of ≥0.006% for human monocytes, a range that is well below what is commonly encountered in most cellular-mediated assays.Of the three methods used in the estimation of SGI in the ADCI assay, the tri-colour and microscopy results were the most similar, consistent with the comparable lower limits of parasitaemia detection. The tri-colour and microscopy both gave generally higher SGI estimates compared to mitotracker red staining alone. This may reflect the systematic underestimation of parasitaemia by mitotracker red staining due to the poor separation between uRBCs and early ring stages of the parasite as demonstrated here. A further elegant output available with the current technique is the ability to quantify the percentage of dead and/or compromised parasites in bioassays, which could potentially be very insightful as has been shown here for the ADCI assay. In addition, the data presented here (Figure 6D) demonstrate the sensitivity of the tri-colour technique to also detect culture specific day-to-day variations in bioassays.

## Conclusion

A tri-colour flow cytometry technique, which is sensitive and versatile and allows for accurate quantification of both live and dead (and/or compromised) parasites as well as leucocytes in bioassays was developed. This method could be very useful for malaria drug testing assays and antibody functional assays such as the ADCI assay and GIA.

## Abbreviations

CPO: Coriphosphine-O
PNIG: Pool of non-exposed immunoglobulin G
PHIG: Pool of hyper immune immunoglobulin G
NHS: Normal human serum
ADCI: Antibody dependent cellular inhibition
GIA: Growth inhibition assay
SGI: Specific growth-inhibition index.
